# Empathy Levels Among Healthcare Professionals: An Asian Multi-professional Cross-Sectional Study

**DOI:** 10.7759/cureus.53750

**Published:** 2024-02-07

**Authors:** Song He, Rehena Sultana, Devanand Anantham, Huey Peng Loh, Jamie X Zhou, Joo Ying Tang, Mabel Sim, Tracy Carol Ayre, Kok Yong Fong, Kok Hian Tan

**Affiliations:** 1 Department of Obstetrics and Gynaecology, KK (Kandang Kerbau) Women's and Children's Hospital, Singapore, SGP; 2 Centre for Quantitative Medicine, Duke-NUS (National University of Singapore) Medical School, Singapore, SGP; 3 Department of Respiratory and Critical Care Medicine, Singapore General Hospital, Singapore, SGP; 4 Institute for Patient Safety & Quality, SingHealth Duke-NUS (National University of Singapore) Academic Medical Centre, Singapore, SGP; 5 Division of Supportive and Palliative Care, National Cancer Centre Singapore, Singapore, SGP; 6 Division of Nursing, Singapore General Hospital, Singapore, SGP; 7 Department of Rheumatology and Immunology, Singapore General Hospital, Singapore, SGP

**Keywords:** singapore, allied health, physicians, nurses, healthcare professionals, empathy

## Abstract

Background: The aim of the study was to measure empathy in healthcare professionals in Singapore and to compare the scores between the different professions: doctors, nurses, and allied health professionals.

Methods: An online survey questionnaire was conducted using the Jefferson Scale of Empathy (JSE) from July 2019 to January 2020. The total JSE score was calculated and compared among the different groups. Multiple linear regression was performed to assess predictors of total empathy scores for groups with statistically lower scores.

Results: The survey was completed by 4,188 healthcare professionals (doctors (n=569, 13.6%), nurses (n=3032, 72.4%), and allied health professionals (n=587, 14.0%)) out of the 9,348-strong survey population, with a response rate of 44.8%. The study revealed a mean empathy score (SD) of 103.6 (15.6) for the cohort. The mean empathy score (SD) was 112.3 (14.7), 101.3 (15.2), and 107.0 (15.0), respectively for doctors, nurses, and allied health professionals. These were statistically significantly different among the groups (p< 0.0001), with nurses scoring significantly lower than either doctors (p< 0.0001) or allied health professionals (p< 0.0001). Multiple linear regression showed that age < 30 years old, male gender, Malay ethnicity, and working in a hospital setting were associated with significantly lower empathy scores in the nursing group.

Conclusion: Nurses in Singapore had significantly lower empathy scores compared to doctors and allied health professionals. Further research on the underlying causes should be undertaken and measures to improve empathy among Singapore nursing staff should be explored and implemented.

## Introduction

Empathy in healthcare refers to a cognitive attribute that involves the ability to understand patients’ experiences and the perspective and ability to communicate this understanding [1]. Empathy is a multidimensional construct that can be expressed in various ways, including verbal and non-verbal communication, supportive gestures, active listening, and providing emotional comfort. Empathy is an important characteristic to possess for healthcare professionals. It has been found to improve communication in the healthcare setting and improve clinical outcomes [2-5], such as improved glycated haemoglobin (HbA1c) results in diabetic patients [5], and shortened duration and reduced severity of common cold [4]. Studies have also shown that a higher empathy level is associated with better patient satisfaction [3,6,7], physician satisfaction [8], and a reduction in malpractice litigation [9-11]. As such, understanding and fostering empathy among healthcare professionals is important.

There is limited data from Singapore on empathy in healthcare. A study that included 446 doctors in residency training found that residents in Singapore recorded a mean (SD) of 104.9 (13.2) on the Jefferson Scale of Empathy (JSE) [12]; this was lower than the reported JSE scores of United States (US) physicians with a mean (SD) score of 120 (15.6) [1]. Another study on 881 medical students showed that the empathy score among medical students in Singapore was, according to Ren and colleagues, lower when compared to their counterparts from the US, and higher compared to those from other Asian countries [13]. There has been no study on empathy levels across all healthcare professionals in Singapore. The Resilience in Academic Medicine (RAM) Survey was launched in July 2019 in SingHealth (Singapore Health Services) to measure the empathy level of all healthcare professionals. SingHealth is Singapore’s largest public healthcare cluster that includes general hospitals, speciality centres, community hospitals, primary care, and SingHealth headquarters (SHHQ). The aim of this paper is to describe the findings of the RAM survey and to compare the empathy scores among the different groups of healthcare professionals in Singapore: doctors, nurses, and allied health professionals.

## Materials and methods

Study design and participants

The RAM Survey was launched on July 18, 2019, by the SingHealth Duke-NUS Institute for Patient Safety & Quality (IPSQ). It was circulated for six months till January 24, 2020. The survey invitation was sent to SingHealth staff with corporate email accounts. It was conducted in SingHealth institutes including four general hospitals (Singapore General Hospital, Changi General Hospital, KK Women's and Children's Hospital, Sengkang General Hospital), five speciality centres (Singapore National Eye Centre, National Cancer Centre Singapore, National Heart Centre Singapore, National Dental Centre Singapore, National Neuroscience Institute), three community hospitals (Bright Vision Community Hospital, Outram Community Hospital, Sengkang Community Hospital), primary care (10 polyclinics), and SingHealth Headquarters (SHHQ). The online survey was hosted on the cluster’s secured intranet and internet platforms. The survey is self-explanatory and was self-administered. All questions required mandatory responses. The survey questions can be found in the Appendices. The participants were categorized into three clinical groups, with the number of sampled populations as follows: Medical (doctors and dentists; n=1,239, 13.3%), Nursing (nurses; n=5,893, 63.0%), and Allied Health (pharmacists, physiotherapists, dieticians, and other allied health professionals; n=2,216, 23.7%) [14]. This yielded a total survey population of 9348. 

Ethical considerations

The study was reviewed and granted exemption by the SingHealth Centralised Institutional Review Board (CIRB) under the category of Anonymous Educational Tests, Surveys, Interviews or Observation (CIRB Reference number: 2019/2495). Survey participants were informed about the purpose and scope of the study through the various publicity platforms including electronic publicity banners and email announcements and at the start of the survey. The study participants were also assured of confidentiality throughout the study process. Survey data was stored in a password-encrypted database and access to the database was limited to authorized study members.

Survey instruments

Demographics

Demographic information of participants such as age group, gender, ethnicity, marital status, profession, years of working experience, etc. were collected as part of this study (Appendix A).

JSE-Healthcare Professional Version (JSE-HP)

The JSE-HP was used in this study as a measurement of the empathy scores of study participants (Appendix B). JSE, a widely used validated tool for measuring empathy, was created by Hojat and colleagues at the Jefferson Medical College in Philadelphia [15]. The JSE-HP version was designed specifically for physicians and other healthcare professionals [1]. The JSE-HP is a 20-item questionnaire that assessed three domains of empathy, including Perspective Taking (PT), Compassionate Care (CC) and Walking in the Patient’s Shoes (WIPS) [1,16]. The items are measured using a seven-point Likert scale, with a score range of 20-140 and a higher score indicates a higher level of empathy [1]. Of the 20 items, 10 (Questions 1, 3, 6-8, 11, 12, 14, 18, and 19) are reversely scored (i.e., Strongly Agree = 1, Strongly Disagree = 7), and the remaining 10 items are directly scored on their Likert weights (i.e., Strongly Disagree =1, Strongly Agree = 7). Evidence to support JSE’s internal consistency [1,15,17,18], and validity, including construct validity [1,15,17], predictive validity [19], and convergent validity [17], has been reported for healthcare professionals. It has also shown a good correlation with other measures of empathy such as the Interpersonal Reactivity Index [20]. There are published findings on the validity and reliability of JSE in medical students and doctors from Asian countries including Singapore [13,21-24]. 

Statistical analysis

Sociodemographic data and baseline characteristics of study participants were summarized as percentages for all variables (all were categorical variables). The mean (SD) of JSE was calculated for the whole cohort and for different health professional groups and compared among the groups. Similarly, the mean (SD) of the three domains of JSE (PT, CC, WIPS) were calculated for the entire cohort and for different groups and compared among the groups. Multiple linear regression was performed to assess predictors of total empathy scores for groups with statistically lower scores. SAS (Statistical Analysis System) (2011) Version 9.3 (SAS Institute Inc., Cary, North Carolina, US) was used for all statistical analyses. Statistical significance was set at p < 0.05.

## Results

Sociodemographic data and baseline characteristics

The total survey population was 9,348. We obtained 4,188 (44.8%) responses from all groups of healthcare professionals. The proportion of the survey respondents were 569 (13.5%) doctors, 3,032 (72.5%) nurses, and 587 (14.0%) allied health professionals.

Table 1 summarizes the sociodemographic variables and professional experiences of the whole cohort and the different groups of healthcare professionals. Most of the participants (n=2,342, 55.9%) were from the age group of 30-49 years; 84% (n=3,531) were female, 55.2% (n=2,313) were married, 49.3% (n=1,988) were parents, 67.7% (n=2,833) were caregivers taking care of young children less than seven years old, or elderly or disabled family members, 3.1% (n=128) were current smokers, 23.6% (n=989) consumed alcohol regularly, 51.0% (n=2,134) were in healthcare for more than 10 years, 74.4% (n=3,116) were working in a hospital setting at the time of study.

**Table 1:** Sociodemographic variables and professional experiences * Hospitals include Singapore General Hospital, Changi General Hospital, KK Women's and Children's Hospital, Sengkang General Hospital ** Specialty Centres include Singapore National Eye Centre, National Cancer Centre Singapore, National Heart Centre Singapore, National Dental Centre Singapore, National Neuroscience Institute *** Others include Community hospitals, Primary care, and SingHealth Headquarters

	Whole Cohort (N=4188)	Medical (n=569)	Nursing (n=3032)	Allied Health (n=587)
Age (years), n (%)				
< 30	1190 (28.4)	059 (10.4)	0971 (32.0)	160 (27.3)
30-49	2342 (55.9)	396 (69.6)	1593 (52.5)	353 (60.1)
≥ 50	0656 (15.7)	114 (20.0)	0468 (15.4)	074 (12.6)
Gender, n (%)				
Female	3531 (84.3)	297 (52.2)	2764 (91.2)	470 (80.1)
Male	0657 (15.7)	272 (47.8)	0268 (08.8)	117 (19.9)
Ethnicity, n (%)				
Chinese	2391 (57.4)	460 (81.4)	1476 (48.9)	455 (77.6)
Malay	0404 (09.7)	058 (10.3)	0312 (10.3)	034 (05.8)
Indian	0650 (15.6)	007 (01.2)	0580 (19.2)	063 (10.8)
Others	0723 (17.3)	040 (07.1)	0649 (21.5)	034 (05.8)
Marital status, n (%)				
Single	1753 (41.9)	167 (29.3)	1301 (42.9)	285 (48.6)
Married	2313 (55.2)	386 (67.8)	1641 (54.1)	286 (48.7)
Others (divorced, separated, widowed)	0122 (2.9)	016 (2.8)	0090 (3.0)	016 (2.7)
Caregiver, n (%)				
Yes	2833 (67.7)	344 (60.6)	2096 (69.1)	393 (67.0)
No	1354 (32.3)	224 (39.4)	0936 (30.9)	194 (33.0)
Number of children, n (%)				
0	2047 (50.7)	255 (45.5)	1442 (49.7)	350 (60.9)
1-2	1533 (38.0)	223 (39.8)	1131 (39.0)	179 (31.1)
≥ 3	0455 (11.3)	083 (14.8)	0326 (11.2)	046 (08.0)
Smoking, n (%)				
Yes	0128 (3.1)	003 (0.5)	0119 (3.9)	006 (1.0)
No	4060 (96.9)	566 (99.5)	2913 (96.1)	581 (99.0)
Alcohol, n (%)				
Yes	0989 (23.6)	238 (41.8)	0584 (19.3)	167 (28.4)
No	3199 (76.4)	331 (58.2)	2448 (80.7)	420 (71.6)
Years of working experience, n (%)				
< 2	0284 (06.8)	015 (02.6)	0216 (07.1)	053 (09.0)
2-5	0640 (15.3)	065 (11.4)	0451 (14.9)	124 (21.1)
6-10	1129 (27.0)	151 (26.6)	0814 (26.8)	164 (27.9)
11-15	0804 (19.2)	085 (15.0)	0620 (20.4)	099 (16.9)
16-20	0468 (11.2)	092 (16.2)	0307 (10.1)	169 (11.8)
> 20	862 (20.6)	160 (28.1)	624 (20.6)	78 (13.3)
Work setting				
Hospital *	3116 (74.4)	390 (68.5)	2321 (76.6)	405 (69.0)
Specialty Centres **	0443 (10.6)	054 (09.5)	0327 (10.8)	062 (10.6)
Others ***	0629 (15.0)	125 (22.0)	0384 (12.7)	120 (20.4)

Empathy level based on JSE-HP

The study revealed a mean empathy score (SD) of 103.6 (15.59) for the whole cohort. The mean empathy score (SD) was 112.3 (14.67), 101.3 (15.18) and 107.0 (14.99) respectively for Medical, Nursing, and Allied Health. These were statistically significantly different among the groups (p< 0.0001), with nurses scored significantly lower than either doctors (p< 0.0001) or allied health professionals (p< 0.0001) (Table 2). When the different domains of JSE were analysed, nurses had the lowest scores in all three domains. The mean (SD) for PT, CC, and WIPS for nurses was 54.4 (9.35), 37.7 (8.12), and 9.2 (2.76), respectively. These were significantly lower than the mean (SD) for doctors (PT 57.4 (8.27), p< 0.0001; CC 44.0 (6.83), p< 0.0001; WIPS 10.9 (2.39), p< 0.0001) and allied health professionals (PT 56.2 (8.06), p< 0.0001; CC 40.5 (7.89), p< 0.0001; WIPS 10.3 (2.58), p< 0.0001) (Table 2).

**Table 2:** Empathy level (Jefferson Scale of Empathy: total score and scores by domains) *Comparisons were made between Medical vs Nursing, and Allied Health vs Nursing.

Professions	Total Score		Perspective Taking		Compassionate Care		Walking in Patient’s Shoes	
	Mean (SD)	p-value*	Mean (SD)	p-value*	Mean (SD)	p-value*	Mean (SD)	p-value*
Nursing (n=3032)	101.3 (15.2)	reference	54.4 (9.4)	reference	37.7 (8.1)	reference	9.2 (2.8)	reference
Medical (n=569)	112.3 (14.7)	< 0.0001	57.4 (8.3)	< 0.0001	44.0 (6.8)	< 0.0001	10.9 (2.4)	< 0.0001
Allied Health (n=587)	107.0 (15.0)	< 0.0001	56.2 (8.1)	< 0.0001	40.5 (7.9)	< 0.0001	10.3 (2.6)	< 0.0001
Whole cohort (n=4188)	103.6 (15.6)	-	55.1 (9.1)	-	38.9 (8.2)	-	9.6 (2.8)	-

Predictors of empathy - nursing

Table 3 summarizes the multiple linear regression analyses for predictors of total empathy score by JSE for the Nursing group. Age of 30-50 years (coefficient 4.23, p<0.01) and >50 years (coefficient 4.87, p<0.01) were positively related to empathy compared to age <30 years. Male nurses had statistically significant lower empathy level compared to female nurses (coefficient -3.28, p<0.01). Among the different ethnic groups, Chinese had significantly higher empathy compared to Malay (coefficient 1.84, p=0.01), but significantly lower empathy compared to other races as a group (including Filipinos, Burmese, etc.) (coefficient -4.97, p<0.01). There was no statistically significant difference in empathy level between Chinese and Indian nurses. Working in a specialty centre (coefficient 2.19, p=0.01) or other settings including community hospitals and primary care (coefficient 3.18, p<0.01) was associated with a significantly higher empathy score compared to working in a hospital setting. 

**Table 3:** Predictors of total empathy score in the nursing group (N=3032) Ϯ Multivariate regression on variables significant at p<0.20 in the univariate analysis, which included age, gender, race, smoking, years of working experience, and work setting. Ϯ Ϯ Comparisons were made between the categorical variable in the row with the reference group of that variable * Hospitals include Singapore General Hospital, Changi General Hospital, KK Women's and Children's Hospital, Sengkang General Hospital ** Specialty Centres include Singapore National Eye Centre, National Cancer Centre Singapore, National Heart Centre Singapore, National Dental Centre Singapore, National Neuroscience Institute *** Others include Community hospitals, Primary care, and SingHealth Headquarters

Variable	Multivariate analysis Ϯ	
	Coefficient (95% CI)	p-value Ϯ Ϯ
Age (years)		
<30	reference	-
30-50	4.23 (3.05, 5.42)	<0.01
>50	4.87 (3.21, 6.53)	<0.01
Gender, Male vs Female	-3.28 (-5.15, -1.41)	<0.01
Race		
Chinese	reference	-
Indian	-0.17 (-1.97, 1.64)	0.86
Malay	-1.84 (-3.27, -0.41)	0.01
Others	4.97 (3.56, 6.37)	<0.01
Work Setting		
Hospital *	reference	-
Specialty Centres **	2.19 (0.46, 3.92)	0.01
Others ***	3.18 (1.56, 4.80)	<0.01

## Discussion

The RAM Survey was the first study on empathy level across different groups of healthcare professionals in Singapore. With a mean empathy score of 103.6 for the whole cohort, Singapore healthcare professionals appear to have lower empathy scores compared to those from many other countries [1,5,25-30]. It also showed that among the different groups of healthcare professionals in Singapore, nurses had significantly lower empathy scores compared to doctors and allied health professionals in terms of total JSE score and in all three domains of JSE. 

Empathy can be compromised by many factors, such as burnout [31,32], high workload, and time constraints [33-35]. Communication barriers due to language and religious differences, as well as unconscious biases when interacting with patients from different backgrounds could have negative impacts on empathy. In addition, a lower degree of personal well-being has been shown to be negatively associated with empathy levels [36,37]. From an organisational level, a hierarchical and authoritarian institutional culture that focuses more on productivity rather than patient-centred care may make healthcare professionals pressured to demonstrate efficacy and thus undermine the value of empathy [35,38].

Doctors in our cohort had a mean empathy score of 112.3. This was lower than that reported for American physicians. Hojat et al. reported a mean JSE score of 120 for 704 American physicians of various specialities [1]. Another study on physician empathy and clinical outcomes from the US showed a mean JSE score of 122.4 by a group of family physicians [5]. The empathy score of doctors from the current cohort was also lower than that from other Western countries, such as Brazil (mean score = 118.5) [25], Italy (mean score = 115.1) [26], and Spain (mean score = 116) [27]. The empathy level of Singapore physicians, however, was higher compared to some studies from other Asian countries. A mean JSE score of 107 was obtained from 537 residents from China [39], and a mean score of 98.2 was reported from a study of 229 Korean physicians [22]. 

Most studies on the empathy of allied health professionals such as pharmacists are done with students. Few studies have been conducted with practising pharmacists and other allied health professionals such as therapists. A study from Japan showed a mean JSE score of 108.7 from 373 licensed hospital pharmacists [40]. A mean empathy score of 118.5 was reported in a cohort of 123 physical therapists from the US [41]. Another study reported a mean JSE score of 121.2 by mental health therapists in the UK [42]. With a mean JSE score of 107.0, allied health professionals in our population appear to score lower in empathy compared to their counterparts based on limited available studies from other countries.

Studies on empathy of nurses have been done in many countries. The empathy of nurses in Singapore (mean JSE score of 101.3) appears to be lower than most studies reported elsewhere. Fields et al. reported a mean JSE score of 117.2 in American nurses [30]. A study on 1,077 nurses across various specialities from 10 public hospitals in China showed a mean empathy score of 109.8 [43]. Another study from China reported a mean JSE score of 111.50 on 236 nurses from an emergency department [44]. In a study of 660 Taiwanese nurses, a mean JSE score of 110.66 was obtained [28]. Similarly, a United Kingdom (UK) study showed a mean baseline JSE score of 110 among nurses in acute hospital settings [45].

Notably, nurses in our cohort had significantly lower empathy scores compared to both physicians and allied health professionals. This was different from most other available studies. Fields et al. demonstrated no statistically significant difference in the empathy level between physicians (mean JSE = 115.7) and nurses (mean JSE = 117.2) from the US [30]. Another study from Poland showed an average JSE score of 113.06 for physicians and 110.12 for nurses with no statistically significant differences [46]. There is a scarcity of evidence comparing empathy levels between other healthcare professionals such as physicians and pharmacists or other allied health professionals, or between nurses and allied health professionals. In a UK study that compared the empathy level of specialist orthopaedic nurses and therapists, nurses were found to have a lower score of empathy (mean JSE = 111.8) compared to therapists (mean JSE = 115.9) [29].

A few possible causes might explain the lower empathy level of nurses compared to other groups of healthcare professionals in Singapore. First, nurses are usually the first-line patient contact in the Singapore healthcare system, and the nature of the nursing job makes them more exposed to the daily concerns, complaints, and possibly negative emotions of patients and their family members compared to physicians and allied health professionals. Studies have shown that empathy is affected by patients’ behaviours and that the response of healthcare professionals varies according to the emotions expressed by their patients [35]. In fact, dealing with angry, frustrated patients and their families is a recognized part of the nursing job [47]. Being potentially more exposed to difficult and demanding behaviours and emotions from patients and their relatives could affect personal wellbeing [31], which will have a negative impact on the empathy level of nurses [48,49]. Second, nurses in Singapore face challenges such as a lack of social recognition from the general public and lower pay compared to other healthcare professionals such as doctors and pharmacists [50]. A study on factors influencing healthcare career choices among Singaporean students showed that low public perception of the value of nursing as a profession was one of the deterrents to students from applying for nursing in Singapore [50]. In addition, nurses’ salaries were perceived to be low compared to other healthcare professionals; being in a materialistic society like Singapore, this was also shown to be an important deterrent to joining nursing among Singaporean students [50]. In fact, it has been reported that nurses in Singapore are underpaid compared to their counterparts in other Asian countries [47,51]. The demands of nursing work and possible dissatisfaction with salary have also been cited as risk factors for nurses’ intention to quit their jobs [52]. These factors contribute to the shortage of nursing staff in Singapore [50], a problem the country has faced for decades [53], which in turn increases nursing workload and potentially reduces the empathy of nurses indirectly. In addition, a large proportion of practising nurses in Singapore are employed from overseas, a strategy by the Singapore government to counteract the problem of nursing workforce shortage [53]. Statistics from the Singapore Nursing Board showed that foreign nurses constituted 29.5% of total registered and enrolled nurses in Singapore in 2021 [54], and more than 50% of new registered/enrolled nurses were foreigners since 2000 [55]. While the employment of foreign nurses helped to increase the number of nursing staff, other problems including language and social and cultural differences may present as a barrier to communication and patient care and affect empathy [56]. Communication has been shown to be one of the crucial aspects in providing and establishing empathetic care to patients [57].

In view of lower empathy scores of nurses compared to other groups in our cohort, multiple linear regression was performed to assess predictors of total empathy score for nursing. Our results showed that male nurses scored significantly lower in JSE. This is consistent with findings from other studies that showed that female health profession students and practitioners had significantly higher JSE scores compared to their male counterparts [1,15,58]. The age group of 30-50 years and >50 years are both positively related to empathy compared to age less than 30 years. This might be explained by the possible effect of work experience on empathy in healthcare workers. A study from Singapore that explored factors contributing to the development of empathy in a healthcare setting suggested that work experiences could improve emotional maturity, coping strategies, and communication skills, which could have a positive impact on empathy [37]. Another study on Singapore nurses found that older nurses are less likely to experience burnout compared to younger nurses [59], where burnout is a known risk factor that reduces empathy among healthcare professionals [31,32]. We also noticed that Chinese nurses had significantly higher empathy scores compared to Malay nurses, but significantly lower scores compared to other races (including Filipinos, Burmese, etc.). This could be attributed to cultural factors unique to the multi-ethnic Singaporean population. With most of the Singaporean population being Chinese, they comprised the largest proportion of the patient population. Chinese nurses can communicate well with most of our elderly patients who are Chinese and are not conversant in English, whereas there might be communication barriers between Malay nurses and Chinese patients that could negatively affect empathy [56]. Interestingly, nurses from other ethnic groups (that constitute 21.5% of the survey participants from the nursing group) obtained a significantly higher mean empathy score. The majority of these nurses are Filipinos. We postulate that this might be linked to the better working environment and higher nursing pay in Singapore compared to their home countries. Most nurses of Malay ethnicity were from Singapore, while nurses of Chinese, Indian, and other ethnic groups had higher proportions coming from other countries. Finally, nurses who worked in a speciality centre or in other healthcare settings such as community hospitals and primary care centres demonstrated higher empathy compared to those who worked in a hospital setting. One possible reason could be that nurses who worked in hospitals might face more time constraints and stress at work as they needed to deal with more acute and complicated medical issues compared to those who work in other healthcare settings such as primary care, and stress and time constraints are known risk factors of lower empathy [35,60].

Empathy is a skill that can be developed and improved with proper training and education. Studies have suggested evidence for empathy training. For example, a study on Taiwanese nurses showed that individuals who received empathy-related training demonstrated significantly higher empathy scores [28]. Other studies have shown that mindfulness course training is associated with improved empathy in healthcare professionals across different disciplines [61]. Although empathy can be enhanced through training and that interventions to improve empathy are often directed on an individual level, developing and maintaining empathy can be challenging for healthcare professionals if the healthcare system they work at does not support or foster empathetic practices [38]. It is therefore essential for healthcare organizations to create a supportive environment that promotes empathy among their staff and addresses potential factors that have a negative impact on empathy. This could involve providing resources for managing stress and burnout, ensuring sufficient manpower and fair work distribution, promoting work-life balance, offering training in empathy and communication, and fostering a patient-centred culture.

Several strengths and limitations merit attention when interpreting the results of the current study. One strength of this study is that it has the largest sample size of 4,188 compared to the few empathy studies previously conducted in Singapore, which all had less than 1,000 responses [12,13]. There are a few limitations to the current study. First, data was collected using a self-administered questionnaire which could have resulted in information bias as respondents may not provide accurate information to all questions so that they could move on quickly through the survey. Second, the survey response rate of 44.8% (n=4,188) was lower than many other similar studies from other countries [1,22,25,27,28,39]. A possible reason to account for the response rate is that potential participants might have been concerned about being identified as detailed demographic information was collected in the survey form and some survey questions might have appeared personal to them, despite the fact that no respondent identities were collected on the survey platform and all the data were kept anonymous. Thirdly, while the current study was carried out in the largest healthcare cluster in Singapore, the findings may not be fully representative of healthcare professionals in other healthcare clusters and the private sector. Lastly, the survey was conducted before the coronavirus disease 2019 (COVID-19) pandemic and thus reflected the empathy level of healthcare professionals in Singapore in the non-pandemic period. Careful considerations are needed for comparison of studies before, during, and post COVID-19 pandemic.

## Conclusions

Among the different groups of healthcare professionals in Singapore, nurses had significantly lower empathy scores compared to doctors and allied health professionals. As nurses form the largest professional group of Singapore's healthcare workforce and many healthcare outcomes hinges directly or indirectly to their performance, further studies on the underlying causes of the lower empathy level among nursing staff should be undertaken. Measures to improve the empathy level among Singapore nurses should be explored and implemented.

## Figures and Tables

**Figure 1:**
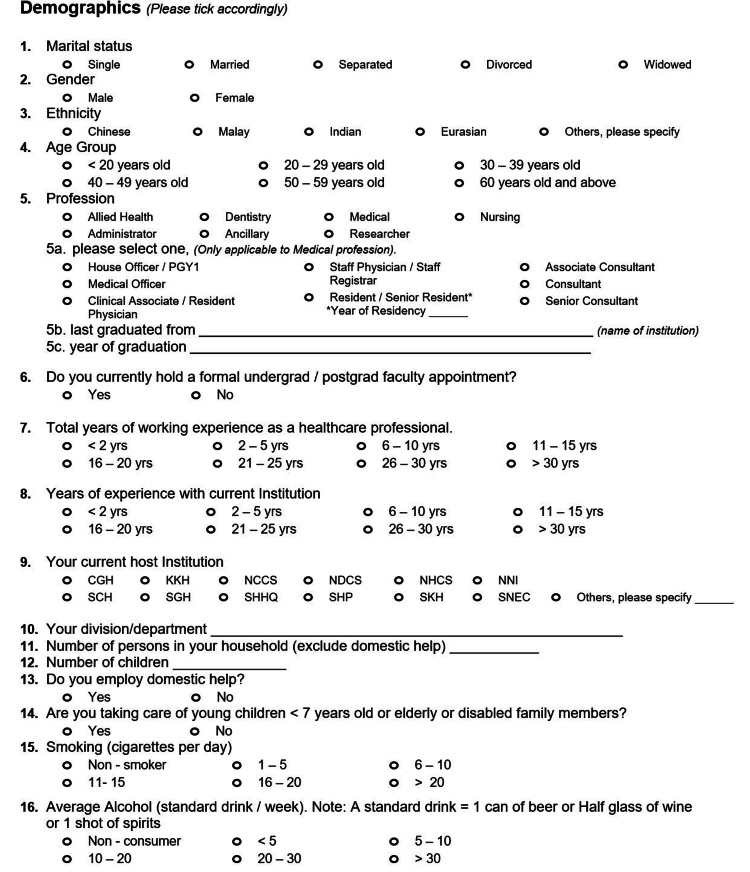
Resilience in Academic Medicine Empathy Survey, Demographics The survey questionnaire on demographics was created by the study team from SingHealth Duke-NUS, Institute for Patient Safety & Quality.

**Figure 2:**
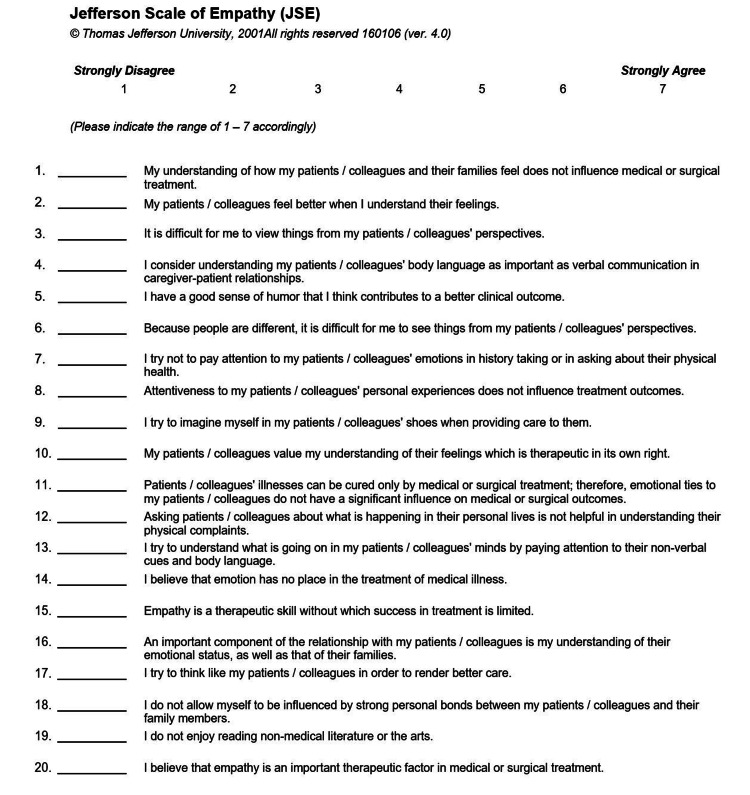
Resilience in Academic Medicine Empathy Survey, Jefferson Scale of Empathy The survey questionnaire on Jefferson Scale of Empathy was purchased from Thomas Jefferson University.

## Appendices

Appendix A

Appendix B
